# Nitrous Oxyd as an Anæsthetic

**Published:** 1866-12

**Authors:** 


### Editorial.

### NITROUS OXYD AS AN ANÆSTHETIC.

THE use of protoxyd of nitrogen, after a protracted dormant period, has recently appeared as an epidemic. That it was so suddenly abandoned, and ether and chloroform substituted for it, is not remarkable, when all the circumstances are duly considered. The non-portability of the gas in sufficient quantities, the lack of apparatus fit to use for a purpose so serious, and the inconvenience in handling such apparatus as was afforded, were well calculated to turn the attention of the profession in other directions. The transitory effect of the protoxyd was also urged as an objection to it, and justly, too, as far as prolonged operations are concerned.

But the nausea, lassitude, and prostration accompanying or following the administration of other anæsthetics, and the fact that, now and again, the wail for the dead was commingled with the fumes of chloroform, have, to some extent, induced anæsthetists to return to their first love, and the protoxyd is rapidly regaining the place which it should have never lost. And, in consistence with the history of the discovery of anæsthesia, in this reform, if reform it be, the Dental profession is taking the lead. Not that Dental surgeons are more progressive or aggressive than general surgeons; but rather that most of their operations, though severe, are brief, and that their patients come to them, while, usually, the general practitioner must go to his, and many of his operations are protracted and tedious, rendering ether, or chloroform more satisfactory. In the use of these, the recumbent posture is very desirable; but this is impracticable with most of the operations of Dental surgery, hence it is but natural that Dentists, above all others, should regard the protoxyd with favor.

But it is possible, that even a good thing may be carried too far. It is acknowledged, by all, that ether and chloroform must be pure. To obtain them thus, in the present state of pharmaceutical chemistry is not very difficult. Reliable manufacturers of the articles are well known. But not so with the nitrous oxyd. While it is fully as important that it be pure, it is more likely to contain impurities than other anæsthetics; and instead of being obtainable from reliable pharmaceutists, the operator has to prepare it for himself. It is not, therefore, to be wondered at, that mistakes occur, and that mischief is done. Indeed, in the present state of affairs, makers and venders of apparatus pressing their wares on the profession, members of the profession buying apparatus because their neighbors have them, and the people demanding freedom from pain in operations, without a willingness to go out of their ordinary haunts to obtain it, the wonder is that more serious consequences do not ensue. True, in the present lack of knowledge, in regard to the proper and legitimate action of the protoxyd, the evils resulting from the inhalation of an impure gas, are nearly always attributed to other causes. They are regarded as merely incidental, even though as legitimate as a burn on the finger, from thrusting it into the fire. A *propter hoc* is regarded as a mere *post hoc*.

Protoxyd of nitrogen, or nitrous oxyd, is usually obtained by the decomposition of nitrate of ammonia. Several considerations in its preparation are worthy of close attention. The nitrate should be absolutely pure. It can now always be thus obtained from reliable chemists. Its composition, as its name indicates, is one equivalent of nitric acid united with one of ammonia. Nitric acid is composed of one equivalent of nitrogen combined with five of oxygen, and its symbol is $NO_5$. Ammonia is formed by the union of one equivalent of nitrogen and three of hydrogen, its formula being $NH_3$. The formula of nitrate of ammonia is, therefore, $NH_3,NO_5$. I am thus minute because I have no right to infer that the readers of the "REGISTER" are all technical chemists. The water of crystallization is not included in the formula, as it has nothing to do with the phenomena of the decomposition.

Now, it will be observed that the three elements composing the nitrate are, when free, all gases. By their native elasticity they have a tendency to resume the gaseous form. This elasticity is increased by heat; and that is the reason that heat alone is able to decompose the nitrate. As the decomposition takes place, new affinities assert themselves. That between oxygen and hydrogen is, perhaps, the strongest known to chemistry. Accordingly, the three equivalents of hydrogen take three of oxygen, and form three equivalents of water. The remaining two equivalents of oxygen take, each, one of the nitrogen, and form the nitrous oxyd, or protoxyd of nitrogen, under consideration. The reactions which take place, when this order of decomposition is perfectly carried out, are clearly set forth by an equatition:

$NH_3,NO_5=3HO+2NO.$

When the nitrate is pure, and properly decomposed, the only results are three equivalents of water and two of nitrous oxyd. But the nitrate is some times contaminated with *sal ammoniac*, in which case, chlorine, a poisonous, suffocating gas, will be commingled with the nitrous oxyd.

But even if the nitrate of ammonia be pure, it may be decomposed by heat, so as to give an impure nitrous oxyd, or even none at all. Nitrogen and oxygen combine in so many proportions, and thus give rise to compounds differing so greatly in their chemical and therapeutic properties, that, to be safe, every step of the process of decomposition must not only be understood, but accurately performed. For example, if the nitrate be decomposed at too high a temperature, instead of nitrous oxyd and water, as in the preceding equation, we may have binoxyd of nitrogen, called *nitric oxyd*, water, and free hydrogen, represented thus:

$(NH_3, NO_5)=2NO_2+HO+H_2.$

Or, at a still higher degree, we may have nitrous acid, ammonia, and oxygen, thus:

$NH_3.NO_5=NO_4+O+NH_3.$

Or, a little higher, the changes are thus represented:

$2\ (NH_3,NO_5)=NO_4+6HO+N_3.$

Any one who can read a chemical formula, will see at a glance, that in any of these decompositions, there is nothing that can sustain respiration for a single inhalation, while an active corrosive poison is present in each of them.

But it is claimed by some, perhaps by many, that these, and all other impurities are removed by washing, that is, by passing the gas through water, on its way from the generator to the receiver. At first sight, this is plausible, and may, therefore, deceive many. But let us examine, as the health, and perhaps the lives of our patients are at issue.

Nitrous acid, $NO_4$, and ammonia, $NH_3$, are both highly soluble in water, and may, therefore, be readily removed by washing. But with the nitric oxyd, or binoxyd of nitrogen, $NO_2$, the case is far different. And, as it is the poison most likely to be formed, it is very important that it be understood.

Nitric oxyd is a colorless, tastless, gas, about as heavy as atmospheric air; it excites violent spasm of the glottis, when an attempt is made to inhale it. Water dissolves only *about* 11 *per cent. of it.* In contact with atmospheric air, it is converted into *nitrous acid*, which, in contact with water, (liquid or vapor) is changed to *nitric acid.* (See Turner's, Graham's, or any standard work on chemistry.)

From the above, it will be seen that nitric oxyd can not be washed out of nitrous oxyd, as water dissolves only 11 per cent. of it, while it dissolves about its own bulk of nitrous oxyd. If generated by itself, or along with nitrous oxyd, it will pass through the washers into the reservoir, or gasometer. Then, if it be inhaled, its effects may be varied. When pure, it can not be inspired, on account of the violent spasm of the glottis produced by it; but diluted with other gases, it may be. Let it be remembered that it is highly corrosive, and its effect on the mucous membrane of the air passages may be appreciated.

But this is not all, nor the half: Nitric oxyd in contact with air, turns to nitrous acid, and this, in contact with water, to nitric acid. As both air and water are always present in the bronchial tubes and cells of the lungs, it is not possible that nitric acid be not formed, if nitric oxyd be inhaled; for the laws of chemical combination are laws of the unchangeable God.

Nor is this all. But if one would boil nitric acid, and cause a patient to inhale the vapor, it would be regarded as quite enough to damn him to infamy, and drive him from the profession. But in that case, the acid would be in its ordinary, or quiescent state, while in the other, its nascent condition gives it greatly increased energy of action.

From the above, it follows that nitric oxyd is *the impurity* to be dreaded in the use of nitrous oxyd as an anæsthetic. The question arises, how is its presence to be avoided. Various attempts have been made to answer this. Protosulphate of iron is sometimes put into the wash-bottles. This salt absorbs nitric oxyd. But unless we know how much we are going to make, while not intending to make any, we can not tell how much of the sulphate to use. Another method is, to admit atmospheric air into the gasometer, the oxygen of which changes the nitric oxyd to nitrous acid, which will be removed by the water; but there is no way to determine how much air to admit. If too much, it dilutes the nitrous oxyd. If too little, it fails to remove the nitric oxyd. In short, the only way is, not to generate any nitric oxyd. And, to attain to this result, we must have apparatus by which the heat can be kept nearly uniform through the entire process of decomposition. Take, for example, a gasometer which requires an hour and a half to fill it. The process may go on exactly right for an hour and a quarter, but, by too high a temperature during the last fifteen minutes, enough of nitric oxyd may be formed to poison the entire contents of the gasometer. No man can give such attention to the process, with ordinary apparatus, as will enable him to *know* that he has not a mixture of nitrous, and nitric oxyds in his gasometer, instead of *pure nitrous oxyd.*

With the uncertainty of the ordinary processes of decomposing nitrate of ammonia, it is not strange that soreness and congestion of the air passages are so frequently observed after the use of the nitrous oxyd. Defective apparatus in the hands of the inexperienced, and those without chemical education, is likely to result imperfectly. A man may be a good anatomist, and a fine operator, without a thorough knowledge of this process; for it is *strictly chemical.* Many are honestly experimenting without sufficient chemical knowledge to be aware of the danger—some, possibly, so far back as to be unwilling to learn. Though a teacher of chemistry for nearly a quarter of a century, I never would, *never did*, and NEVER WILL use a nitrous oxyd apparatus, even for amusement, without the ability absolutely to control the temperature at which the gas is generated. The man of science has no more right to profane the laws of nature than the minister of the gospel has to profane the laws of revelation.

Sprague's apparatus gives us thorough control of the process of decomposition, and there may be others that do, but I have not seen them. If not in existence now, I take it for granted that such soon will be.

But I began, only to write a page; yet the subject is, by no means exhausted. I may refer to it in a future number. W.

